# Multi-omics analysis identifies a role for *Streptococcus suis* MnmE in growth regulation and environmental adaptation

**DOI:** 10.1128/spectrum.01201-26

**Published:** 2026-05-22

**Authors:** Yuxuan Huang, Jing Sun, Jiyu Zhao, Jieyu Bai, Jiyun Chai, Jutao Wang, Yang Zhou, Paul R. Langford, Yueling Zhang, Gang Li

**Affiliations:** 1 State Key Laboratory for Animal Disease Control and Prevention, Division of Bacterial Diseases, Harbin Veterinary Research Institute, Chinese Academy of Agricultural Sciences 111613 , Harbin, China; 2 Section of Paediatric Infectious Disease, Department of Infectious Disease, Imperial College London, St. Mary's Campus 4615, London, United Kingdom; Emory University School of Medicine, Atlanta, Georgia, USA

**Keywords:** *Streptococcus suis*, MnmE, virulence, tolerance, multi-omics analysis

## Abstract

*Streptococcus suis* is a major bacterial pathogen of pigs and a significant zoonotic threat to human health. A critical unresolved question is whether the bacterium can rapidly respond to adverse environmental conditions by regulating the expression of mRNA rich in specific codons, thereby facilitating colonization and infection. MnmE, a highly conserved multi-domain GTPase, plays a key role in tRNA U_34_ modification, which is essential for maintaining translational fidelity across both bacterial and eukaryotic organisms. Compared with wild-type *S. suis*, a Δ*mnmE* mutant displayed growth defects, altered morphology, reduced virulence, impaired environmental tolerance, reduced capsular polysaccharide production, and defective carbohydrate utilization. Omics analyses confirmed perturbations in carbohydrate metabolism, transcription/translation, and ion transport, consistent with phenotypic changes. Notably, mRNA enriched in U_34_ codons did not solely determine protein expression upon loss of U_34_ tRNA modification enzymes, instead revealing a translational bias toward A-ending codons within synonymous codons. Furthermore, we demonstrated that MnmE is required for intracellular metal ion homeostasis by influencing the expression of proteins related to metal ion transport, which in turn affects intracellular reactive oxygen species levels and the ability to survive. These findings highlight that MnmE is indispensable for *S. suis* physiology and pathogenicity.

**IMPORTANCE:**

*Streptococcus suis* is a major swine and zoonotic pathogen. While bacterial tRNA modifications are known to fine-tune translation, their role in coordinating pathogenesis and stress adaptation remains poorly characterized. Here, we demonstrate that the tRNA-modifying GTPase MnmE is essential for *S. suis* virulence and environmental resilience. Through integrated multi-omics and phenotypic analyses, we show that MnmE deletion cripples carbohydrate metabolism, disrupts metal homeostasis, and attenuates virulence by globally dysregulating stress-response networks and virulence effectors. Crucially, we reveal that U_34_ codon enrichment in mRNAs does not predict translational outcomes when MnmE is absent, uncovering new complexity in post-transcriptional regulation. This work advances our understanding of tRNA modification as a node linking translational fidelity to metabolic adaptation and pathogenicity, extending beyond model microorganisms.

## INTRODUCTION

*Streptococcus suis* is a Gram-positive bacterial pathogen responsible for substantial economic losses in the global swine industry (1). It causes diverse porcine diseases, including meningitis, septicemia, endocarditis, and arthritis (2). Critically, *S. suis* is also an emerging zoonotic agent; human infections can lead to severe clinical manifestations such as streptococcal toxic shock syndrome (STSS). Outbreaks in humans are frequently associated with direct contact with infected pigs or contaminated pork products (3).

Southeast Asia represents a high-prevalence region for human *S. suis* infection, in part attributable to local dietary practices. For example, China reported major outbreaks in 1998 and 2005 (4, 5), and human cases continue worldwide (6–9), underscoring the persistent public health threat (10). Based on capsular polysaccharide (CPS) antigenicity, *S. suis* is classified into 29 serotypes (11), with serotype 2 being the most clinically prevalent in both pigs and humans (5). However, significant virulence heterogeneity exists even among strains of the same serotype or within clonally related lineages (12, 13), complicating disease management.

During infection, *S. suis* must adapt dynamically to host-imposed stresses, including oxidative bursts, nutrient limitation, and defend against host-derived antimicrobial mechanisms (14, 15). While these stress response systems are vital for bacterial survival, their molecular regulation in *S. suis* remains poorly understood. One protein that has a role in the stress response is MnmE, a conserved homodimeric GTPase (formerly TrmE), which catalyzes tRNA wobble uridine (U_34_) modifications essential for the accurate decoding of NNA and NNG codons (16). Such tRNA modifications can selectively reprogram protein synthesis (17), preventing translational frameshifting, ensuring fidelity in stress-responsive protein production, and thereby facilitating bacterial adaptation (18, 19).

Emerging evidence across prokaryotes and eukaryotes highlights tRNA modifications as a universal “molecular coping mechanism” to survive environmental perturbations (20). In response to stress, cells dynamically reprogram tRNA modifications—termed “tRNA epitranscriptome reprogramming”—to prioritize translation of mRNAs enriched in specific codons, so-called “modification-tunable transcripts (MoTTs)”, that encode stress-response or survival proteins. For instance, in *Mycobacterium bovis*, hypoxia induces the conversion of tRNA-Thr-CGT wobble modifications, promoting translation of dormancy-associated genes with ACG codon enrichment (21). In contrast, *Saccharomyces cerevisiae* uses Trm9-dependent U_34_ modifications to enhance translation of DNA damage response genes with AGA/GAA codons (22).

Beyond its canonical translational role, MnmE exhibits pleiotropic functions in bacterial pathogenesis: while MnmE is essential in *Escherichia coli* (23), deletion attenuates virulence in *Salmonella enterica* (24), *Pseudomonas syringae* (25), *Aeromonas hydrophila* (26), and *Streptococcus pyogenes* (27). MnmE was recently implicated in modulating arginine metabolism in *S. suis* (28), suggesting broader regulatory roles in its physiology.

Given the conserved role of tRNA modifications in stress-induced translational reprogramming, we hypothesized that MnmE-mediated U_34_ modification might act as a core regulator of *S. suis* adaptation to host and environmental stresses by coordinating codon-biased translation of key survival genes.

Here, we investigated the function of MnmE in the *S. suis* serotype 2 strain 05ZYH33 by constructing an isogenic Δ*mnmE* strain and comparing it with the wild type (WT). Phenotypic analyses revealed *mnmE*-dependent defects in growth kinetics, stress tolerance, metal homeostasis, and virulence. Multi-omics profiling demonstrated systemic dysregulation of cell cycle progression, division, virulence factor expression, and carbohydrate/inorganic ion metabolism, alongside perturbations in transcriptional and translational networks. Another key finding was that U_34_ codon enrichment alone did not fully determine proteomic outcomes under modification defects; other uncharacterized sequence features likely collaborate with codon composition to dictate translational bias. Collectively, our results establish MnmE involvement in coordinating stress adaptation in *S. suis* through integrated control of protein synthesis and metabolic flux, advancing the understanding of translational regulation in bacterial pathogenesis.

## RESULTS

### MnmE plays a crucial role in maintaining growth and viability

**Fig 1:**
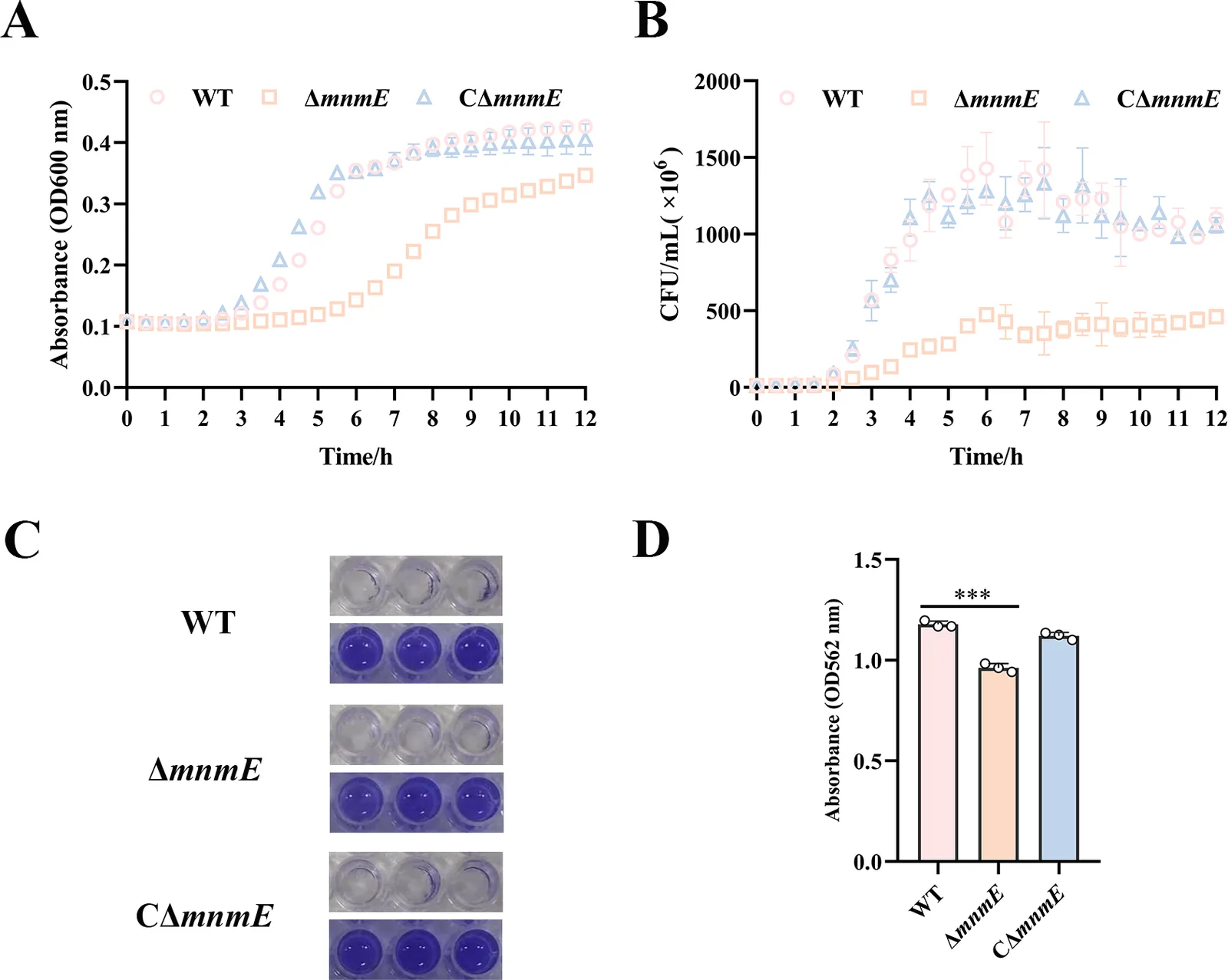
Effects of MnmE on bacterial growth characteristics. (**A**) Growth curves of the wild-type (WT), Δ*mnmE* (mutant), and CΔ*mnmE* (complemented) strains. Bacterial growth was monitored by measuring the optical density at 600 nm (OD_600_) at various time points. (**B**) Cell viability of the WT, Δ*mnmE*, and CΔ*mnmE* strains. The number of colony-forming units per milliliter (CFU mL⁻¹) was determined at the indicated different time points. The cultures were serially diluted with fresh medium and spotted onto THA plates. Shown is a representative assay from at least three independent experiments. Error bars represent the standard deviations from biological replicates. (**C and D**) Biofilm assay of the WT, Δ*mnmE*, and CΔ*mnmE* strains. Biofilm formation was assessed in 96-well polystyrene microtiter plates. The plates were stained with crystal violet. The OD_562_ of the biofilm was measured after 12-h incubation. Error bars indicate standard deviations. Statistical significance was determined by an unpaired *t*-test using GraphPad Prism 8 (***P < 0.001).

To explore the influence of MnmE on cell growth and viability, we monitored the growth of the 05ZYH33 (WT), Δ*mnmE* (mutant), and CΔ*mnmE* (complemented) strains. The growth rate of Δ*mnmE* was distinctly slower than that of the WT (Fig. 1A), further validated by colony-forming unit (CFU) counting, where the viability of the Δ*mnmE* mutant was lower than that of the WT strain (Fig. 1B). Notably, the complementation strain CΔ*mnmE* restored the growth defect, underscoring the significant role of *mnmE* in the growth of *S. suis*. Conventional 96-well assays in polystyrene plates were used to assess the ability of strains to form biofilms. Compared with the WT and CΔ*mnmE*, Δ*mnmE* was impaired in biofilm formation. These findings suggest that MnmE is involved in *S. suis* biofilm formation.

### MnmE is associated with bacterial morphological changes

**Fig 2:**
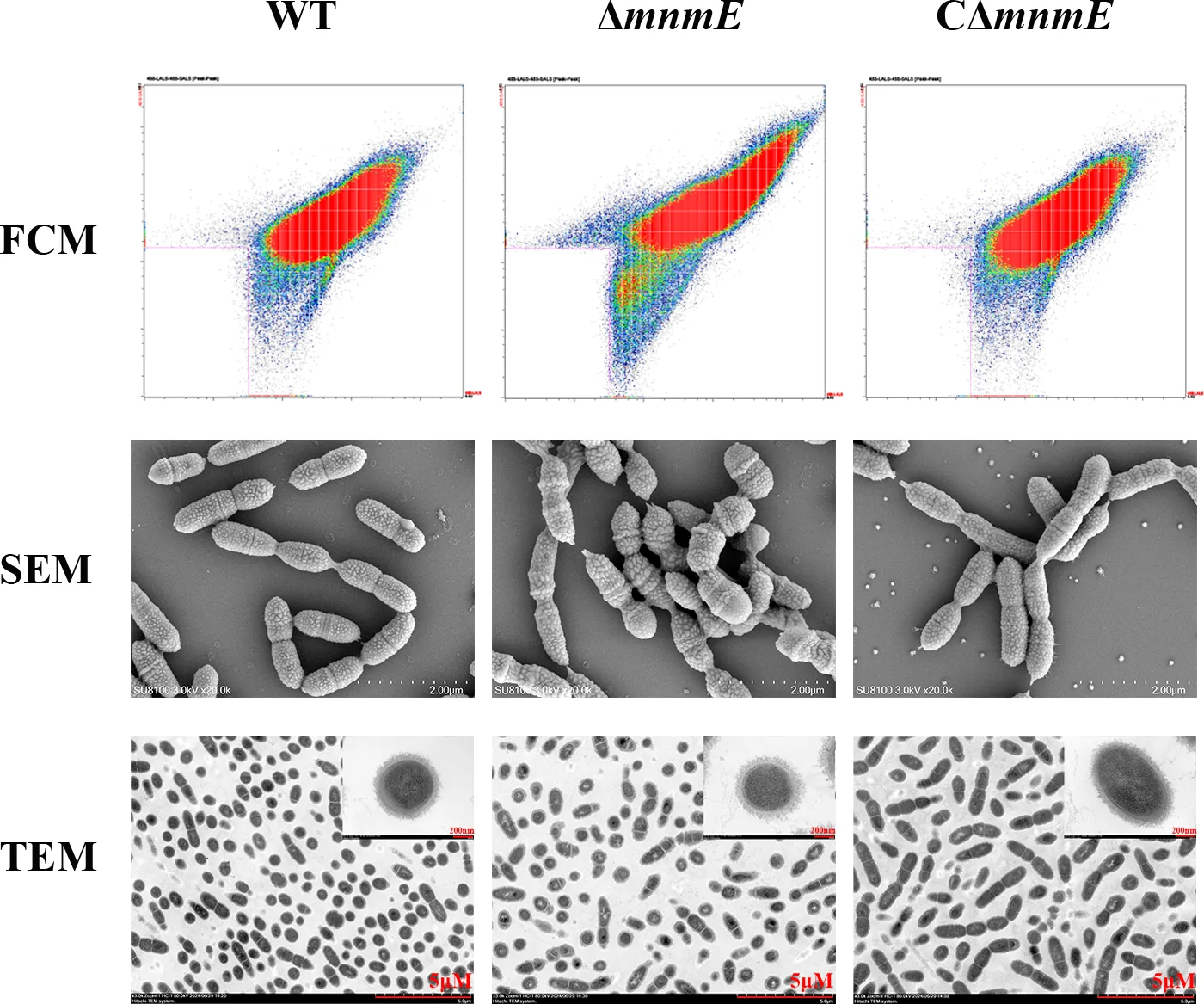
Effects of *mnmE* on the morphology wild-type (WT), Δ*mnmE* (mutant), and CΔ*mnmE* (complemented) strains. Morphological characteristics of WT, Δ*mnmE*, and CΔ*mnmE* strains observed via flow cytometry (FCM), scanning electron microscopy (SEM), and transmission electron microscopy (TEM). In the FCM images, blue, green, and red indicate increasing bacterial counts from few to many. The *x*-axis represents forward scatter (FSC), which is positively correlated with cell size; the *y*-axis represents side scatter (SSC), which is positively correlated with the complexity of intracellular granular structures. In the TEM and SEM images, red arrows mark the morphological abnormalities observed in the Δ*mnmE* strain.

**Fig 3:**
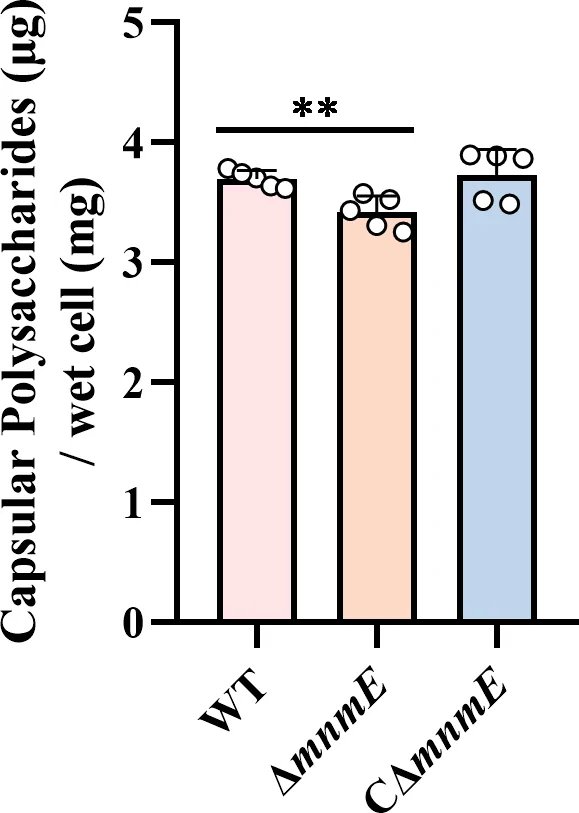
Quantification of capsular polysaccharide (CPS). The CPS content of wild-type (WT), Δ*mnmE* (mutant), and CΔ*mnmE* (complemented) strains were determined by using a modified phenol-sulfuric acid assay. Statistical significance was determined by an unpaired *t*-test using GraphPad Prism 8 (**P < 0.01).

To further explore the effect of MnmE on the morphological and structural characteristics of *S. suis*, multiple microscopic techniques were employed. Flow cytometry (FCM) revealed two distinct subpopulations in the Δ*mnmE* mutant based on forward scatter (FSC-A, indicative of cell size) and side scatter (SSC-A, indicative of internal granularity/complexity), which were not present in the WT (Fig. 2). This suggests heterogeneity in cell size and internal composition in the mutant. Scanning electron microscopy (SEM) results (Fig. 2) further supported those from FCM. The WT exhibited uniform morphology and smooth surfaces, while the mutants showed varying sizes, rough surfaces, and apparent accumulation of an extracellular polymeric substance, likely polysaccharide at the septation sites (red arrows, Fig. 2). Transmission electron microscopy (TEM) showed that the capsule of the mutant tended to be thicker but more loosely bound to the cell wall (Fig. 2). Next, we quantified the CPS content of the WT and mutant strains. The results showed that the CPS content in the mutant was significantly lower than that in the WT (Fig. 3), consistent with the scanning electron microscopy results. Thus, the *mnmE* gene plays a vital role in maintaining the morphology of *S. suis*.

### MnmE affects the *in vitro* tolerance of *S. suis* to different stress factors

**Fig 4:**
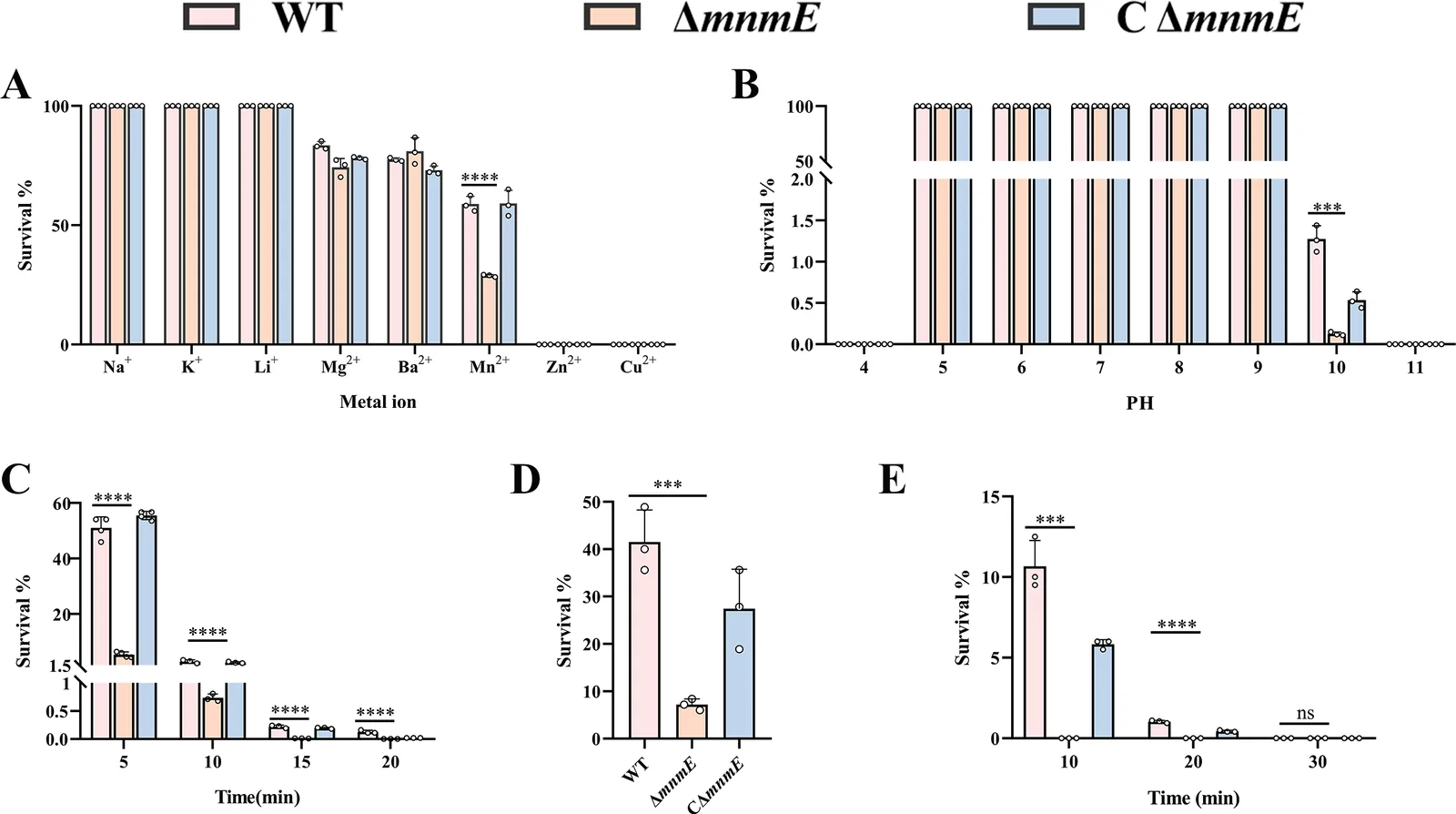
Impaired stress tolerance of the strains. (**A**) Metal ion tolerance. The survival of WT, Δ*mnmE*, and CΔ*mnmE* strains when exposed to various metal ions. (**B**) pH tolerance. The tolerance of the strains to different pH levels. (**C**) Thermal tolerance at 50°C. The ability of the strains to withstand thermal stress at 50°C. (**D**) Oxidative stress tolerance. The resistance of the strains to oxidative stress. (**E**) Tolerance to glutaraldehyde and deciquam solutions. Each experiment was replicated three times, and representative data are shown. Statistical significance was determined by an unpaired *t*-test using GraphPad Prism 8 (***P < 0.001; ****P < 0.0001; ns, not significant).

To determine the impact of *mnmE* deletion on the tolerance of *S. suis* to different stress factors, we compared the survival rates of WT and mutant strains before and after treatment under the same stress conditions. The results indicated that the *mnmE* gene influenced the *in vitro* tolerance of *S. suis* to various stress factors. When exposed to different metal ions, the Δ*mnmE* mutant showed no significant difference in survival compared with the WT in the presence of KCl, NaCl, LiCl, MgCl_2_, BaCl_2_, CuCl_2_, or ZnCl_2_ (Fig. 4A). However, it was more sensitive to MnCl_2_, suggesting a potential role of the *mnmE* gene in regulating Mn^2+^ transportation. The Δ*mnmE* mutant was less tolerant at pH 10 than the WT (Fig. 4B). All strains demonstrated similar survival capabilities under other pH gradient conditions. Under thermal stress at 50°C, the Δ*mnmE* mutant also exhibited lower tolerance compared with the WT (Fig. 4C); its survival rate dropped sharply after 5 min of treatment. As the incubation time increased, the survival rate of all strains decreased accordingly. The Δ*mnmE* mutant also had reduced oxidative stress tolerance (Fig. 4D); its survival rate decreased nearly fourfold under the same treatment conditions. When exposed to the common swine farm disinfectants glutaraldehyde and deciquam, the Δ*mnmE* mutant showed significantly reduced tolerance (Fig. 4E). The WT strain survived a 20-min treatment, with a small number of bacteria remaining viable. However, extending treatment to 30 min eliminated all viable WT bacteria. Overall, the absence of the *mnmE* gene impairs the survival ability of *S. suis* under various stress conditions.

### Impact of the *mnmE* gene on multiple virulence-related traits of *S. suis*

**Fig 5:**
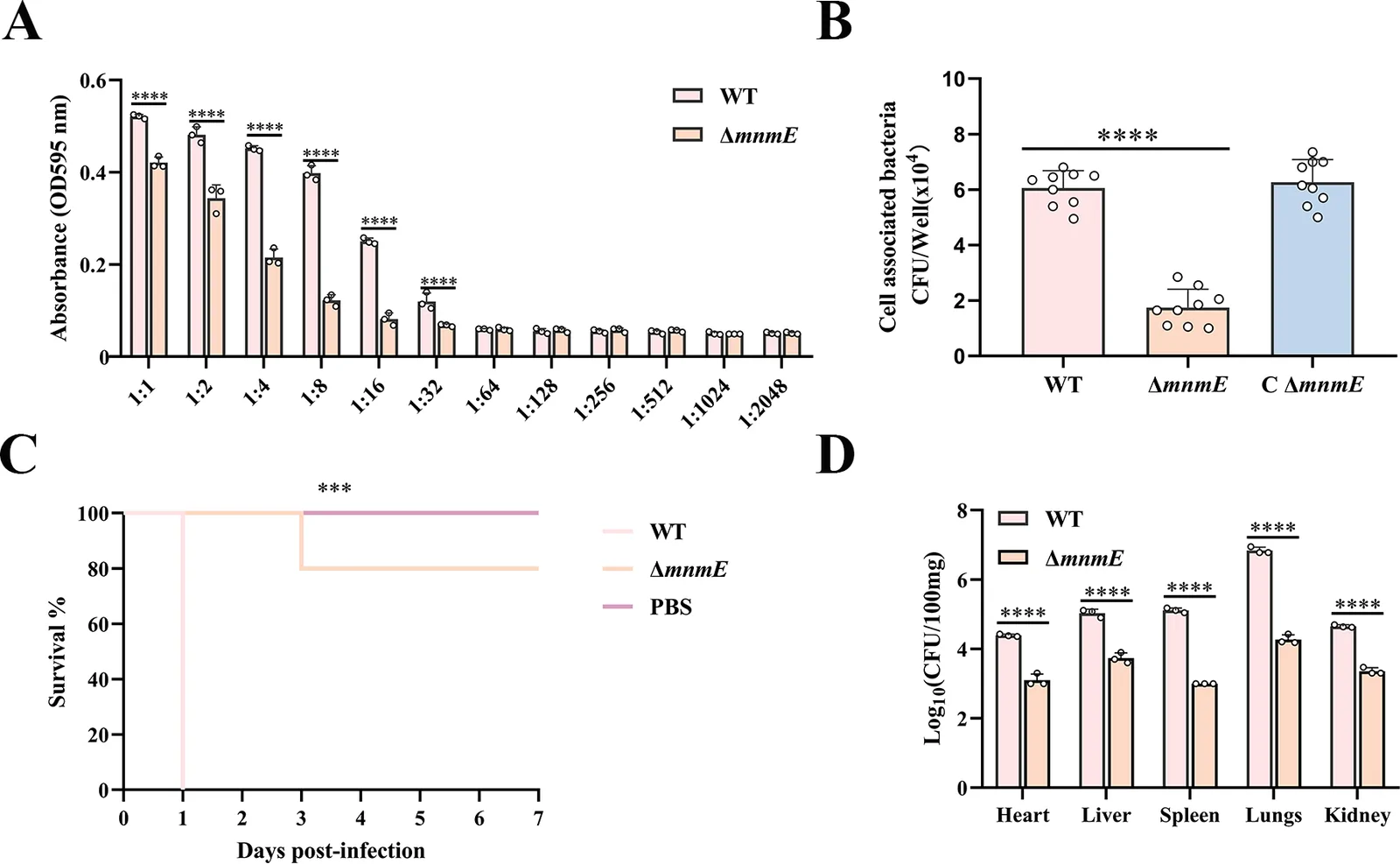
MnmE contributes to *S. suis* pathogenicity *in vivo* and *in vitro*. (**A**) Assessment of hemolytic activity. The hemolytic activity of the mutant strain Δ*mnmE* toward sheep red blood cells was significantly reduced compared to the WT strain between 1:1 and 1:32 ratios. (**B**) Bacterial association to NPTr cells. Compared with the WT and CΔ*mnmE*, the mutant strain Δ*mnmE* showed a significantly reduced cell-association with NPTr cells. (**C**) Mouse survival curves after experimental infection. Significant differences in survival rates between different groups are shown. (**D**) Bacterial loads in different mouse organs. Data are presented as CFU/mL. Statistical significance was determined by an unpaired *t*-test using GraphPad Prism 8 (***P < 0.001, ****P < 0.0001). All data are represented as mean ± SEM of triplicate samples.

To determine the effect of *mnmE* on the virulence of *S. suis*, we co-incubated WT and Δ*mnmE* mutant strains with Newborn pig tracheal (NPTr) cells and simultaneously conducted challenge assays in mice. There was significantly less hemolysis of sheep red blood cells by the *mnmE* mutant compared with WT (1:1–1:32) (Fig. 5A). This indicated that deletion of the *mnmE* gene substantially reduced the hemolytic activity of *S. suis*. In terms of cell-associated capacity, experiments showed that the binding of the WT to NPTr cells was approximately three times that of the Δ*mnmE* mutant (Fig. 5B), demonstrating that the absence of the *mnmE* gene significantly weakened the ability of *S. suis* to associate with pig respiratory epithelial cells.

The virulence of *S. suis* was also affected by the *mnmE* gene. In a mouse infection model, the LD50 of the Δ*mnmE* mutant was higher than that of WT, suggesting reduced pathogenicity. During the infection experiment, all mice infected with the WT developed severe symptoms and died within the first day. In contrast, mice infected with the Δ*mnmE* mutant had milder symptoms, with only one out of five dying during the 7-day observation period (Fig. 5C). The bacterial load in vital organs (hearts, livers, spleens, lungs, and kidneys) of mice infected with the Δ*mnmE* mutant was lower than that in mice infected with the WT (Fig. 5D). Overall, these results suggest that the lack of the *mnmE* gene attenuates the virulence of *S. suis*.

### Molecular mechanisms underlying virulence attenuation and stress sensitivity in the Δ*mnmE* mutant

To explore the potential molecular mechanisms underlying phenotypic changes, we systematically compared the differences in transcriptome and proteome between WT and *mnmE* mutant strains. Compared with the WT strain, the Δ*mnmE* strain exhibited 504 differentially expressed genes (DEGs) (199 upregulated and 305 downregulated) (Table S2) and 288 differentially expressed proteins (DEPs) (120 upregulated and 168 downregulated) (Table S3). Cross-omics comparison revealed that 17.3% (87/504) of the DEGs showed concordant regulation at the protein level, with 24 co-upregulated and 63 co-downregulated. (Fig. S4). We randomly selected several DEGs identified by RNA-seq and validated them using RT-qPCR to independently verify the reliability of the RNA-seq data (Fig. S5).

Transcriptomic analysis showed critical vulnerabilities in stress response networks (Fig. S5A). Heat shock resilience was compromised (Fig. 4C) through suppressed expression of chaperones (*grpE*/*dnaK*) and the alternative sigma factor *ecfA2* (SSU05_0742), whose encoded proteins are essential for protein folding and stress-induced gene expression. Oxidative defense mechanisms faltered (Fig. 4D) due to downregulation of peroxide resistance regulators (*perR*/*dinF*) and free-radical scavengers (e.g., *dps*). Concurrently, proteomic analysis revealed that deletion of *mnmE* in *S. suis* triggered extensive molecular dysregulation (Fig. S5B). Some virulence-associated genes (*uppS* [SSU05_1964], *pepDA* [SSU05_1387], *ndk* [SSU05_0839], *msmX* [SSU05_1907], *OTC* [SSU05_0626], and *slo* [SSU05_1403])—whose products are involved in cell wall biosynthesis, nutrient acquisition, and host tissue damage-were all significantly downregulated (Table S5). This linked MnmE to virulence attenuation and compromised stress tolerance. This kind of coordinated suppression of virulence factors provides a mechanistic basis for the reduced hemolytic activity, impaired epithelial adhesion, and attenuated pathogenicity in mice of the Δ*mnmE* mutant compared to WT (Fig. 5A through D).

Consistent with the role of MnmE in stress adaptation, the Δ*mnmE* mutant exhibited enhanced sensitivity to MnCl_2_ relative to the WT strain (Fig. 4A). Quantitative proteomic analysis revealed significant downregulation of TroA and TroB—proteins implicated in metal ion (including Mn^2+^) uptake and homeostasis—whereas FeoA and FeoB, which mediate ferric (Fe^2+^) uptake, were upregulated in the mutant. Based on these observations, we hypothesized that dysregulation of the Mn^2+^/Fe^2+^ homeostasis ratio contributes to increased intracellular reactive oxygen species (ROS) levels in the Δ*mnmE* mutant, rendering it more susceptible to Mn^2+^ stress.

**Fig 6:**
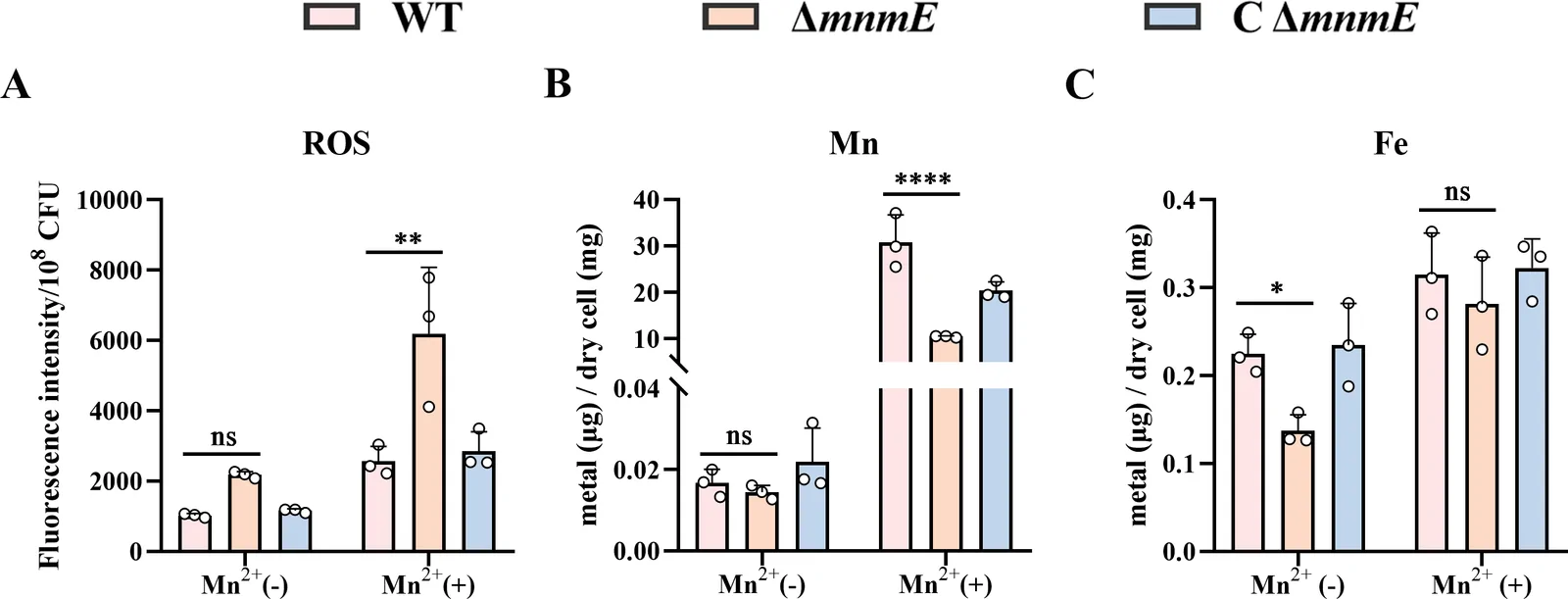
The effect on ROS levels and the concentrations of metal (Mn, Fe) *in vivo*. (**A**) Reactive oxygen species (ROS) levels of different strains under conditions with/without Mn^2+^ treatment. (**B**) Manganese (Mn) content of the different strains under conditions with/without Mn^2+^ treatment. (**C**) Iron (Fe) content of the different strains under conditions with/without Mn^2+^ treatment. Statistical significance was determined by a two-tailed *t*-test using GraphPad Prism 8 (*P < 0.05; **P < 0.01, ****P < 0.0001; ns, not significant).

To test this hypothesis, we measured intracellular ROS levels and the concentrations of Fe^2+^ and Mn^2+^ *in vivo* under Mn^2+^-exposed and unexposed conditions. Our results demonstrated that deletion of MnmE significantly increased intracellular ROS levels compared with the WT strain. Notably, elevating the extracellular Mn^2+^ concentration further exacerbated intracellular ROS accumulation in the Δ*mnmE* mutant, a phenotype directly linked to the mutant’s reduced viability (Fig. 6A). Parallel analysis of intracellular metal ion concentrations confirmed that exogenous Mn^2+^ exposure disrupted metal ion homeostasis in the Δ*mnmE* mutant. In the absence of Mn^2+^ stress, intracellular Mn^2+^ levels were comparable between the Δ*mnmE* mutant and the wild-type strain. In contrast, under Mn^2+^ stress, the Δ*mnmE* mutant exhibited significantly lower intracellular Mn^2+^ levels than the WT strain. Concomitantly, intracellular Fe^2+^ levels were elevated in bacterial cells under Mn^2+^ stress; specifically, Fe^2+^ concentrations in the Δ*mnmE* mutant increased to levels comparable to those of the WT strain (Fig. 6B and C). This shift in metal ion concentrations resulted in a higher Fe^2+^/Mn^2+^ ratio in the Δ*mnmE* mutant, and such disruption of intracellular metal ion homeostasis further impaired the mutant’s viability.

### Multi-omics revealed a central role for MnmE in carbohydrate metabolism

Integrated multi-omics analyses indicated that *mnmE* deletion globally constrained carbohydrate utilization in *S. suis*, establishing its role as a key regulator of metabolic networks. Transcriptomic and proteomic analyses identified significant enrichment of downregulated genes and proteins involved in galactose metabolism (Fig. 6A and B), with gene set enrichment analysis (GSEA) at transcript (Fig. 6C) and translation (Fig. 6D) levels revealing coordinated suppression of additional central pathways: amino sugar and nucleotide sugar metabolism, starch and sucrose metabolism, and glycolysis/gluconeogenesis. These pathways are essential for energy production and biosynthesis of cell wall components, explaining the mutant’s growth defects and morphological abnormalities (Fig. 2 and 3). Metabolomic profiling validated reduced metabolic flux through these pathways, with perturbations in key intermediates, such as 2-phosphoglycerate, UDP-N-acetylglucosamine, and D-galactose-1-phosphate (Fig. 5E). While proteomic changes were less pronounced than transcriptional ones-likely due to post-translational buffering (29), integrated pathway mapping (combining transcriptomic and metabolomic signatures) exposed broad attenuation across metabolic nodes, confirming functional impairment (Fig. 5F). Critically, functional validation through carbon source utilization assays confirmed the inability of the Δ*mnmE* mutant to catabolize diverse substrates, including D-maltose, D-raffinose, D-galactose, and N-acetyl-D-glucosamine (Table 1). This defect was exacerbated by downregulation of components of the phosphotransferase system (PTS) (e.g., *agaFV*, *celA*, *manXY*), which mediate uptake and phosphorylation of carbohydrates. Together, these findings indicate that *S. suis* MnmE coordinates the translation of carbohydrate utilization-related enzymes to optimize carbohydrate utilization, linking translational fidelity to energy harvesting and metabolic fitness.

**TABLE 1:** MnmE affects fermentation of different carbohydrates in *S. suis^a^*

Carbohydrate	WT	Δ*mnmE*	Carbohydrate	WT	Δ*mnmE*
D-Amygdalin	–	–	Lactose	+	–
D-Sorbitol	–	–	Salicin	+	–
D-Ribose	–	–	Cyclodextrin	–	–
D-Maltose	+	–	D-Galactose	+	–
D-Mannitol	–	–	N-Acetyl-D-glucosamine	+	–
D-Raffinose	+	–	Amylopectin	+	–
D-Trehalose	+	–	Sucrose	+	+^w^
D-Mannose	+	–	Methyl-β-D-glucopyranoside	+	–
D-Xylose	–	–			

^*a*^ “+”: Positive; “+^w^”: Weak positive; “–”: Negative result.

### The frequency of U_34_ codons does not dictate protein fate

**Fig 7:**
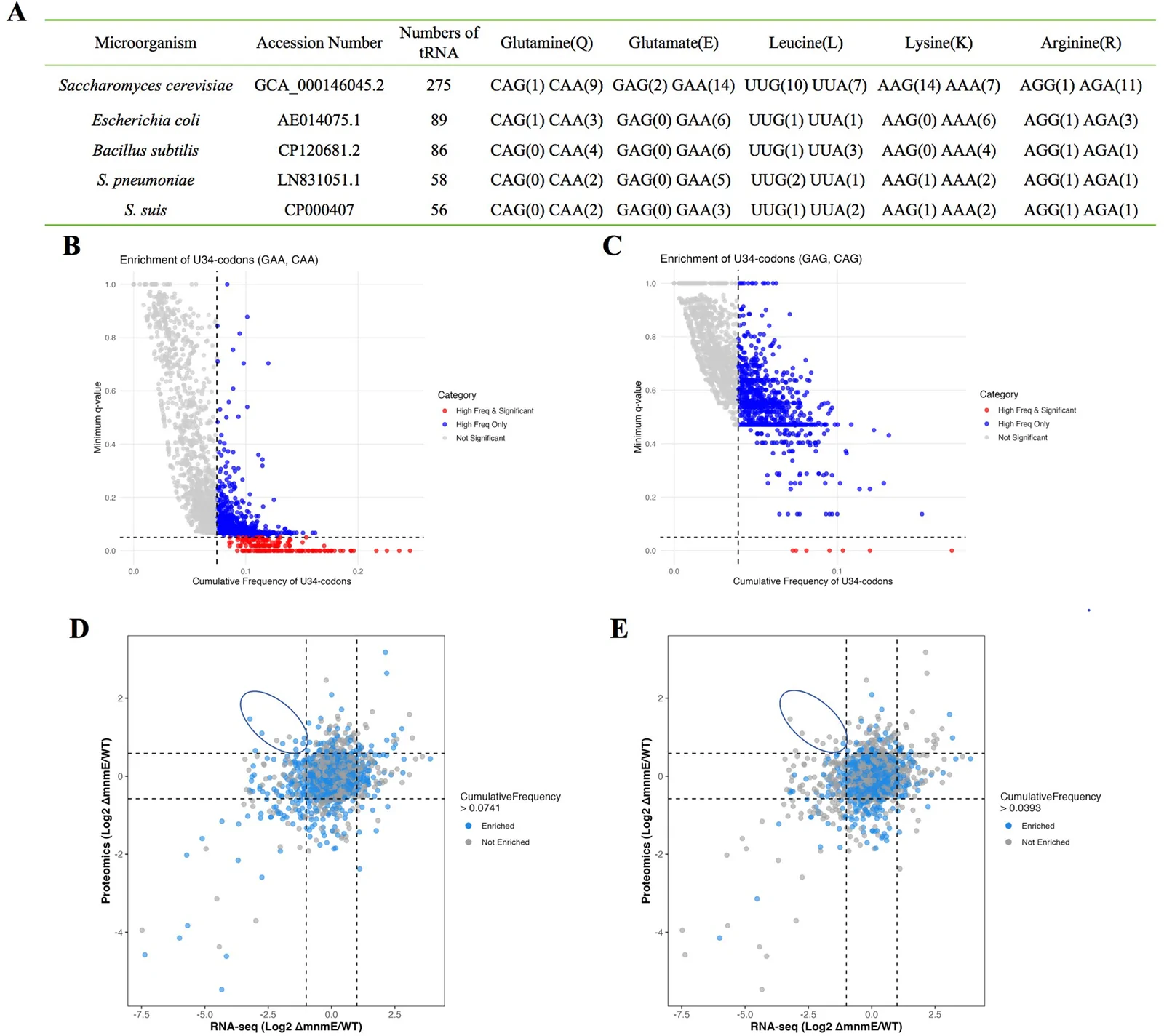
mRNA codon content is a poor predictor of translation bias upon MnmE loss. (**A**) Analysis of the number of tRNAs encoded in the genomes of *S. suis*, *S. pneumoniae*, and three other model microorganisms, as well as the number of tRNAs decoding amino acids of the five two-family codons. (**B and C**) CAA, GAA or CAG, GAG codon frequency, and *q* value were computed for each gene. Blue dots represent genes enriched in the five U_34_ codons (cumulative frequency >0.0741 for CAA/GAA and >0.0393 for CAG/GAG, respectively). (**D and E**) Plots of mRNA vs protein expression ratios are shown. Points in blue are enriched in U_34_ codons (CAA, GAA [**D**] or CAG, GAG [**E**]).

To investigate whether the deletion of *mnmE* leads to translational bias due to differences in U_34_ codon content, we characterized the quantity and types of tRNAs within the *S. suis* genome. The findings revealed that the genome harbored 56 genes encoding 31 distinct tRNA species. Notably, while two tRNA copies decoded the codon Gln^CAA^, no tRNAs were identified for its synonymous codon Gln^CAG^. Similarly, three tRNA copies existed for Glu^GAA^, yet none were encoded for its synonymous codon Glu^GAG^ (Fig. 6A). These observations contrast with codon usage frequencies (CAG:17.1‰, GAG: 22.2‰) in *S. suis*. Subsequently, we conducted a genome-wide analysis of U_34_ codon groups (GAA/CAA vs GAG/CAG; Fig. 6B and C). Using a *q*-value threshold of <0.05 and cumulative frequency >0.0741/0.0393, we statistically defined codon enrichment across all coding sequences. Unexpectedly, while downregulated proteins showed significant U_34_ codon enrichment in mRNAs, most genes with enriched U_34_ codons also were unchanged in protein expression (Fig. 7D and E), suggesting that U_34_ codon content alone cannot predict protein levels in the Δ*mnmE* mutant. However, we found that for the existing DEPs (also differentially expressed at the transcriptional level) in *S. suis*, there is a preference in the use of synonymous codons: the proportion of codons ending with A (67%) (Fig. 7D) is significantly higher than that of codons ending with G (27%) (Fig. 7E). This preference for A-ending synonymous codons differs from the codon usage frequency determined by the overall GC content (41.85%) and third letter GC content (38.53%) of the *S. suis* genome, indicating a specific, non-random translational bias under MnmE loss. Taking proteins that are downregulated at the transcriptional level but upregulated at the translational level as an example, it appears that the reduced enrichment of codons ending with G serves as a prerequisite for their upregulation at the translational level.

## DISCUSSION

The role of MnmE in coordinating stress adaptation aligns with the conserved “tRNA reprogramming” mechanism described across kingdoms (20). This study establishes the tRNA-modifying GTPase MnmE as a critical regulator of *S. suis* physiology and pathogenicity. Through comprehensive phenotypic characterization and multi-omics analysis of an isogenic Δ*mnmE* mutant, we demonstrate that MnmE is indispensable for normal growth kinetics, maintenance of cellular morphology, environmental stress tolerance, metal ion homeostasis, efficient carbohydrate utilization, and full virulence. These defects in the Δ*mnmE* mutant correlate with widespread molecular dysregulation affecting pathways central to cell division, stress response, virulence factor expression, ion transport, and metabolism.

U_34_ modification is known to affect the stress response in *Mycobacterium tuberculosis*, where U_34_ modification of tRNA-Thr enables translation of hypoxic survival genes (21), and *Escherichia coli*, where MnmE-dependent U_34_ modifications regulate stress-induced translation of redox defense proteins (23). Our findings extend this paradigm to *S. suis*, where MnmE-mediated U_34_ modification acts as a molecular switch to prioritize translation of stress-response and metabolic genes—likely via codon-biased mechanisms conserved in pathogenic bacteria. In addition, the role of MnmE in carbohydrate metabolism—particularly its regulation of galactose and glycolytic pathways—adds a new dimension to tRNA modification-mediated stress adaptation.

We acknowledge the prior study by Gao et al. (28), which established the importance of MnmE for growth, pathogenicity, and arginine metabolism in *S. suis* strain SC19. Our current study, using strain 05ZYH33, builds upon this foundation to provide a mechanistic dissection of how MnmE influences bacterial physiology. Here, we have additionally documented novel roles for MnmE in biofilm formation, metal ion homeostasis, oxidative stress tolerance, and carbohydrate utilization, aspects not investigated previously by Gao et al. (28). More importantly, by integrating multi-omics analyses, we provide a mechanistic explanation for these functions through multi-omics integration, e.g., linking metal sensitivity to TroA/B expression changes and ROS imbalance.

**Fig 8:**
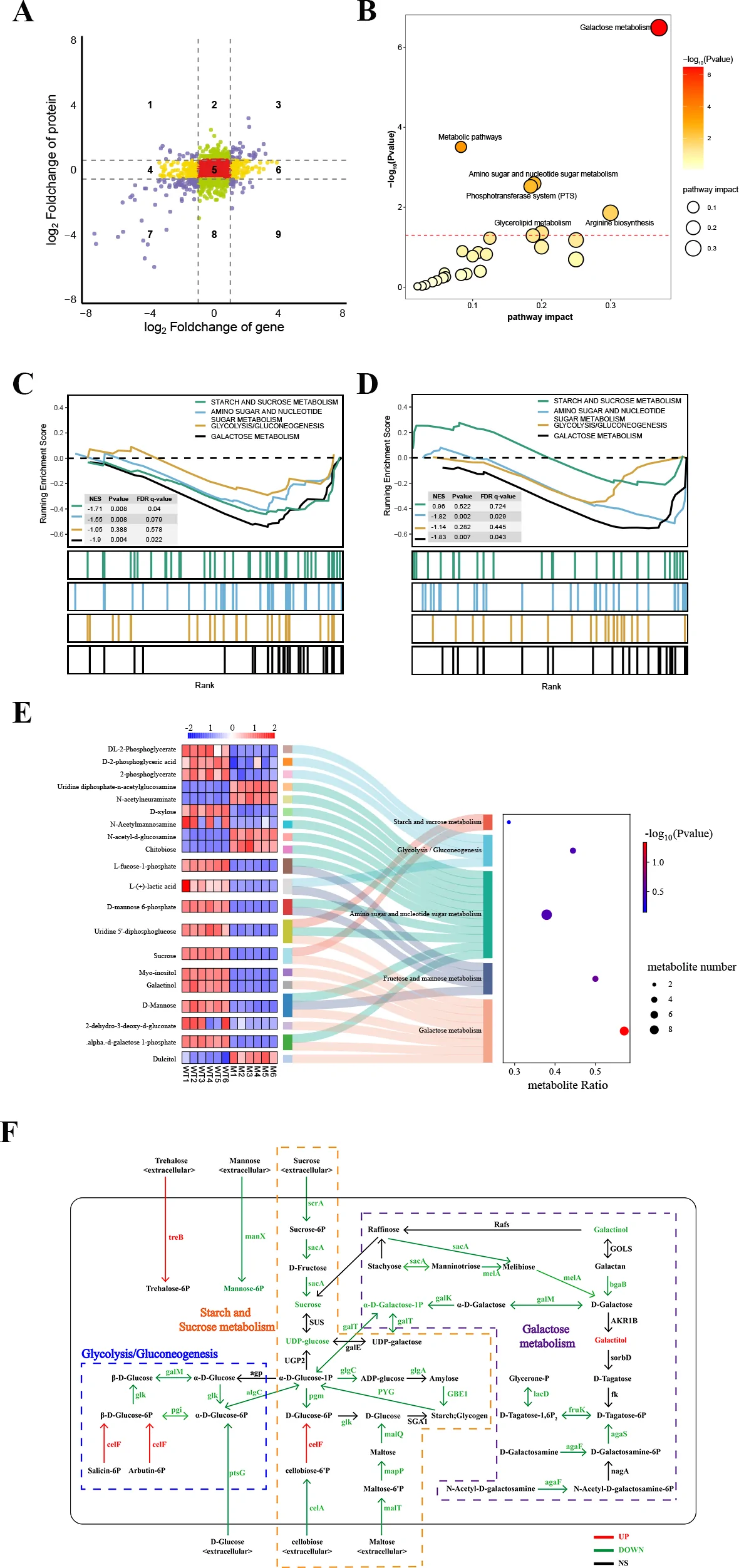
Integrated multi-omics analysis comparing WT and Δ*mnmE* strains. (**A**) Nine-quadrant plot depicting mRNA vs protein expression ratios (WT/Δ*mnmE*). Data points are color-coded: purple (significant changes in both mRNA [DEGs: adjusted *P*-value < 0.05, |log_2_FC| > 1] and protein [DEPs: adjusted *P* value < 0.05, |FC| > 1.5]); green (significant protein changes only); yellow (significant mRNA changes only). (**B**) Integrated analysis of genes and proteins corresponding to quadrant 7 in panel **A**. Pathway impact: number of differential genes annotated to a pathway or number of background genes annotated to that pathway. It reflects how strongly the pathway is over-represented in the differential gene set; larger values indicate greater enrichment. (**C and D**) Gene Set Enrichment Analysis (GSEA) of transcriptomic (**C**) and proteomic (**D**) data for key carbohydrate metabolism pathways: galactose metabolism, amino sugar and nucleotide sugar metabolism, starch and sucrose metabolism, and glycolysis/gluconeogenesis. The peak of the curve indicates the point in the ranked gene list (ordered from most positively to most negatively associated with the mutant phenotype) where the gene set is most enriched. A peak on the left side (high rank) indicates that genes in the set are generally upregulated in the mutant (i.e., higher in Δ*mnmE* vs WT). A peak on the right side (low rank) indicates the gene set is generally downregulated. In this study, all pathways shown have their peaks on the right with negative normalized enrichment scores (NES), indicating that genes in these carbohydrate metabolism pathways are coordinately downregulated at both transcriptional and translational levels in the Δ*mnmE* mutant, consistent with the mutant’s carbohydrate utilization defect. (**E**) KEGG pathway enrichment analysis of differentially expressed metabolites (DMs: VIP ≥ 1, *P*-value < 0.05). (**F**) Schematic representation of partial carbohydrate metabolism pathways, integrating data from DMs and DEGs.

A key insight is the disconnect between U_34_ codon enrichment and protein expression in Δ*mnmE*, which mirrors observations in eukaryotes (20, 30). Such findings align with emerging insights into the complexity of translational regulation by tRNA modifications. As highlighted by Gustilo et al. (19), tRNA modification defects do not simply reduce translation of all U_34_ codon-enriched mRNAs; instead, additional factors—including protein sequence features and dynamic changes in tRNA modification stoichiometry—dictate translational outcomes. In *S. cerevisiae*, for example, Trm9-dependent U_34_ modifications promote translation of AGA/GAA-enriched genes only when they encode proteins with specific hydrophilic motifs, which prevents aggregation during translational pausing (30). Similarly, in *Mycobacterium bovis*, starvation-induced shifts in tRNA pools indicate that codon bias alone is insufficient to predict translation efficiency; instead, coordination between tRNA abundance and modification status determines protein output (31). Indeed, when analyzing the codon usage frequency in *S. suis*, we observed that CAG and GAG are abundant in the genome. However, interestingly, no cognate decoding tRNA genes were identified. A similar phenomenon occurs in several other prokaryotic model bacteria, such as *E. coli* and *B. subtilis* (Fig. 8A) but is absent in *S. cerevisiae*. Thus, U_34_ modification may be particularly critical for prokaryotes to enhance the ability of tRNA to decode synonymous codons.

In *S. suis*, the observed translational bias toward A-ending synonymous codons in the *ΔmnmE* mutant cannot be simply explained by genomic GC content, suggesting that uncharacterized sequence features (e.g., aggregation-prone motifs, chaperone-binding sites [30]) or tRNA modification dynamics collaborate in regulating translation.

In this study, multi-omics comparison of WT and its isogenic Δ*mnmE* mutant was conducted after their growth in standard laboratory conditions, i.e., aerobically in THB plus serum. In earlier studies of codon preference translation, bacteria were typically grown under stress conditions (20–22). Whether the preferential use of A-ending synonymous codons by *S. suis* would be further amplified under stress conditions remains to be determined. Future studies using codon-swapped reporter systems or motif-directed mutagenesis could clarify how these factors interact with U_34_ modifications to shape the proteome (22). Our study expands the complexity of post-transcriptional control in *S. suis*, highlighting that tRNA modifications act as part of a broader network integrating codon usage and protein biogenesis.

Finally, the conservation of MnmE functions in pathogens (24, 27) and its role in regulating virulence factors makes it a promising therapeutic target. In *S. suis*, targeting MnmE could impair both stress tolerance and metabolic adaptation, reducing its ability to colonize hosts and cause disease. This study extends the understanding of the responses in growth and metabolism to the absence of tRNA modifications in bacteria beyond model microorganisms. It also explored the molecular coping mechanisms through initial analysis of preferential codon usage: a relatively unexplored area of bacterial pathogenicity. Future work will determine the detailed molecular mechanisms underlying MnmE function, particularly its role in coordinating codon-biased translation of key regulators during host infection.

## MATERIALS AND METHODS

### Bacterial strains, media, and culture conditions

*S. suis* serotype 2 strain 05ZYH33 (WT), preserved in our laboratory (32), was used as the parental strain. Strain 05ZYH33 was routinely cultured in Todd-Hewitt broth (THB; Becton Dickinson, USA) supplemented with 5% Donor Equine Serum (Cytiva, China) at 37°C with shaking at 180 rpm. For solid media, THB was supplemented with 1.5% agar (Coolaber, China) and 5% Donor Equine Serum (Cytiva, China) = Todd Hewitt Agar (THA) plates. Antibiotics were added to the media as required: erythromycin at a concentration of 4 µg/mL was used to select for mutant strains, and spectinomycin at 100 µg/mL was used to select for complemented strains. *E. coli* MC1061F- (33) (WeiDi, China) was used for plasmid propagation and transformation, was grown in Luria-Bertani (LB) broth (Coolaber, China) or on LB agar plates at 37°C.

### Construction of *mnmE* gene deletion and complemented strains

The *mnmE* gene deletion strain (Δ*mnmE*) was constructed using a previously described markerless gene-deletion protocol (34). The primers were designed based on the genome sequence of *S. suis* strain 05ZYH33 (GenBank: CP000407) and are listed in Table S1. For the construction of the complemented strain, a *mnmE* gene fragment was amplified by PCR using primers CM-F/CM-R. The amplified fragment was cloned into the plasmid pSET2, resulting in the creation of the recombinant plasmid pSET2-*mnmE*, which was first propagated in *E. coli* MC1061F-. Subsequently, pSET2-*mnmE* was transferred into the Δ*mnmE* mutant through peptide-induced transformation. The complemented strain CΔ*mnmE* was selected on THA plates based on its resistance to spectinomycin. To confirm successful complementation, the *mnmE* gene and its flanking regions were amplified by PCR using two pairs of primers.

### Determination of growth characteristics

The growth characteristics of WT, Δ*mnmE*, and CΔ*mnmE* were assessed by monitoring changes in optical density at 600 nm (OD_600_) using a plate reader (Envision, PerkinElmer, USA). To initiate the experiment, each strain was first cultured to the mid-exponential growth phase. Subsequently, the cultures were diluted 1:100 in fresh THB supplemented with 5% Donor Equine Serum. Aliquots of 200 µL from each diluted culture were then transferred to a microplate, and the OD_600_ was measured every 30 min over a 12-h period at 37°C. This method allowed for the continuous and precise monitoring of bacterial growth dynamics under controlled conditions.

### Quantitative biofilm assay

The microtiter plate biofilm assay, a static assay, is highly effective for investigating the early stages of biofilm formation (35). First, overnight cultures were diluted 100-fold with THB. Then, 200 μL of the diluted culture was added to each well of a sterile 96-well polystyrene microtiter plate (Costar 3599, Corning, NY, USA). The plate was incubated at 37°C for 48 h. After incubation, the wells were gently washed with 200 μL of water to remove non-firmly adherent cells. The excess water was removed by inverting the plate several times onto fresh paper towels. Subsequently, each well was filled with 100 μL of 0.1% crystal violet solution and incubated at room temperature for 15 min. After removing the crystal violet solution, the wells were re-washed with 200 μL of water and dried in an incubator at 37°C for 30 min. Finally, 100 μL of 33% acetic acid was added to each well, and the absorbance was measured at 562 nm.

### Morphology assays

The morphological characteristics of the WT, Δ*mnmE*, and CΔ*mnmE* strains were examined using three distinct methods. For flow cytometry (FCM, Apogee) analysis, samples were washed three times with PBS and then directly analyzed using the FCM. For scanning electron microscopy (SEM, SU8100, Hitachi) analysis, samples were fixed with Electron Microscope Fixative (Servicebio, China) for 2 h at room temperature. Following fixation, cell pellets were dehydrated through a graded ethanol series (30% to 100%) for 10 min each, washed in isoamyl acetate for 10 min, and then subjected to vacuum drying for 6 h. A 5-µL aliquot of the dried sample was applied to aluminum foil and sputter-coated with platinum before examination under the SEM. For transmission electron microscopy (TEM, HT7800, Hitachi) analysis, samples were fixed with Electron Microscope Fixative for 2 h at room temperature, followed by post-fixation with 1% osmium tetroxide at 4°C for 1–2 h. After rinsing with PBS for 15 min, samples underwent graded ethanol dehydration (30% to 95%) for 15 min each, followed by treatment with 100% ethanol, pure acetone, and epoxy embedding medium for 20 min each. The samples were then embedded in epoxy resin and dried at 70°C for 12 h. Thin sections (70–90 nm) were cut using an ultramicrotome and examined under the TEM. These analyses were performed on cells harvested during the mid-exponential phase of growth.

### Quantification of capsular polysaccharides

CPS was quantified with minor modifications according to a published protocol (36). Cells were grown to stationary phase, harvested at equal biomass, centrifuged (10,000 × *g*, 10 min, 4°C), and washed with PBS, and 0.5 g wet cells were resuspended in 10 mL of 0.1 M glycine buffer (pH 9.2) containing 100 mg lysozyme (Beyotime, China). After 24-h incubation at 37°C to lyse the cell wall, the suspension was centrifuged, and the supernatant was treated with 10 mg DNase I and 5 mg RNase A (Takara, Japan) at 37°C for 1 h, followed by 10 mg Proteinase K (Takara, Japan) at 55°C for 2 h. CaCl_2_ was then added to a final concentration of 0.1 mol/L, and the solution was stirred for 1 h. Cold ethanol was added to 25% (vol/vol), the mixture incubated at 37°C for 3 h, and precipitates removed by centrifugation (8,000 × *g*, 20 min, 4°C). The supernatant was adjusted to 80% (vol/vol) cold ethanol to precipitate polysaccharides at 4°C. After centrifugation, the pellet was vacuum-dried and redissolved in 3-mL deionized water. CPS content was determined by a modified phenol–sulfuric acid method: 600 μL sample was mixed with 300 μL 5% phenol, and 1.5 mL 93% sulfuric acid was added rapidly. The mixture was vortexed and color allowed to develop at room temperature for 10 min, and the absorbance at 490 nm determined. A calibration curve was prepared with a 1:1:1:1 (by mass) mixture of mannose, rhamnose, galactose, and N-acetylgalactosamine, and CPS concentration was calculated accordingly.

### *In vitro* tolerance assessment

We assessed the tolerance of WT, Δ*mnmE*, and CΔ*mnmE* strains to various environmental stresses by culturing them to the mid-exponential growth phase and subjecting them to various stresses. Mid-exponential cells were harvested, washed three times with 1 mL of PBS buffer, and prepared for subsequent experiments. To evaluate metal ion tolerance, the strains were resuspended in 1 mL 20 mM concentrations of different salts, including KCl, NaCl, LiCl, MgCl_2_, MnCl_2_, BaCl_2_, CuCl_2_, or ZnCl_2_. For pH tolerance testing, the strains were resuspended in 1 mL of PBS with pH levels adjusted from 3 to 11 for 1 h. To determine thermal tolerance, the strains were incubated in a water bath at 50°C for 5, 10, 15, or 20 min. For assessing oxidative stress tolerance, the strains were resuspended in 1 mL of PBS buffer containing 15 mM H_2_O_2_ and incubated at 37°C for 1 h. For glutaraldehyde and deciquam treatment: cells were resuspended in the working solution of glutaraldehyde and deciquam and treated for 10, 20, or 30 min. Following the stress exposures, bacterial suspensions were incubated at 37 °C with 5% CO_2_ for 12 h, and CFUs were determined.

### Hemolytic activity assays

Cultures of *S. suis* strains WT and Δ*mnmE* were cultured in THB until the OD_600_ reached 0.6, washed twice with PBS, and resuspended in fresh THB medium. Aliquots of 200 μL were transferred to a 96-well microplate, twofold serially diluted to a final dilution of 2,048, and 100 μL of 2% sheep red blood cell suspension (Solarbio, China) was added per well. After incubation at 37°C for 2 h, the plate was centrifuged at 1,000 × *g* for 10 min to pellet intact red blood cells. Supernatants (100 μL) were transferred to a new plate, and absorbance at 595 nm was measured using a microplate reader. The experiment was repeated three times with three replicates per sample to ensure reliability.

### Cell-associated assays

Cell-associated assays were conducted according to established protocols (37). NPTr cells were cultured to confluence and then infected with the WT, Δ*mnmE*, and CΔ*mnmE* strains, which were harvested from the mid-exponential growth phase, at a multiplicity of infection (MOI) of 100:1. The infected cells were incubated for 1 h at 37°C in a 5% CO_2_ atmosphere to facilitate bacterial adhesion. Subsequently, unbound bacteria were removed by gently washing the cells three times with PBS. The cells were then lysed by scraping in 1 mL of ice-cold MilliQ water. To quantify the cell-associated bacteria, serial dilutions of the lysates were prepared and plated onto THA plates. The plates were incubated at 37°C for 24 h, and CFUs were determined.

### Mouse infection experiments

To assess the relative pathogenicity of Δ*mnmE* compared with WT, we refined a previously established protocol and performed quantitative analysis of viable bacterial burdens in major organs (38). We used 15 female SPF BALB/c mice (5–6 weeks old), weighing 15 to 16 g, and randomly assigned them into three groups. On the third day post-infection, heart, liver, spleen, lung, and kidney samples were collected from three mice in each group, homogenized, and serial dilutions were plated onto THA plates. The plates were incubated at 37°C for 24 h, and CFUs were determined to quantify the bacterial load in each organ.

### Sample preparation for omics analysis

WT and Δ*mnmE* were grown to the exponential phase (6 h) in THB medium at 37°C in an incubator with 5% CO_2_. Cells were harvested at 4°C, washed twice with ice-cold PBS, and flash-frozen as pellets in liquid nitrogen. Three biological replicates were used for transcriptomic and proteomic analyses. The metabolomics added three more, giving six in total. Subsequently, samples were sent to the Beijing Genomics Institute (BGI)-Shenzhen in China (http://www.genomics.cn/index) in dry ice for sequencing and analysis.

### Identification of differentially expressed genes by RNA-seq analysis

Extracted bacterial RNA was assessed for purity using a Nanodrop 2000 spectrophotometer (Thermo Fisher Scientific, MA, USA), and concentration and integrity of RNA samples were evaluated using a Qubit 3.0 Fluorometer (Thermo Fisher Scientific, MA, USA) and an Agilent 2100 Bioanalyzer (Agilent Technologies, CA, USA), respectively. Library construction was performed using the NEBNext Ultra RNA Library Prep Kit for Illumina (#E7530L, NEB). After library quality was confirmed using the Agilent 2100 Bioanalyzer, sequencing was conducted on an Illumina HiSeq X TEN.

Raw reads were filtered using Fastx_toolkit (version 0.0.14) (http://hannonlab.cshl.edu/fastx_toolkit/index.html) with the following criteria: (i) removal of reads where more than 20% of bases had a quality score (Q) below 15; (ii) removal of reads containing adapter contamination; (iii) removal of reads with more than 5% unknown bases (N). High-quality clean reads were obtained after filtering. Clean reads were aligned to the *S. suis* reference genome using HISAT2 (v2.1.0) (39). HTSeq (v0.11.2) was used to count the aligned reads and generate the raw count matrix, which served as the input for differential expression analysis (40). DEGs were identified using edgeR (3.26.6) (41). The count matrix was normalized using the TMM (Trimmed Mean of M values) method. Genes with |log2[fold change (FC)]|>1 and FDR < 0.05 were considered differentially expressed.

### Identification of differentially expressed proteins by LC-MS/MS

Proteins were extracted from bacterial cells using SDT lysis buffer (4% SDS, 100 mM Tris-HCl, pH 7.6) and quantified using the BCA method. Trypsin digestion was performed using the FASP method (42). Peptides were desalted using a C18 column, lyophilized, and reconstituted in 40 μL of 0.1% formic acid. Peptide concentration was determined by OD_280_, and iRT standard peptides were added. Samples were analyzed by LC-MS/MS in DIA mode on an Astral high-resolution mass spectrometer (Thermo Fisher Scientific, MA, USA). Parameters: positive ion mode; precursor ion scan range: 380–980 m/z; MS1 resolution: 240,000 at 200 m/z; normalized AGC target: 500%; maximum injection time: 5 ms. MS2 used DIA acquisition with 299 scan windows, isolation window of 2 m/z, HCD collision energy of 25 eV, normalized AGC target of 500%, and maximum injection time of 3 ms.

Raw data were processed using DIA-NN software. Parameters: enzyme: trypsin; max missed cleavage sites: 1; fixed modification: carbamidomethyl (C); variable modifications: oxidation (M) and acetyl (Protein N-term). Identified proteins were filtered at FDR <1%. Proteins with FC > 1.5 (upregulated >1.5-fold or downregulated <0.67-fold) and *P*-values < 0.05 (*t*-test) were considered differentially expressed.

### Identification of differentially expressed metabolites

Untargeted metabolite detection was performed using a Vanquish UHPLC system (Thermo Fisher Scientific) with a Phenomenex Kinetex C18 column (2.1 mm × 50 mm, 2.6 μm). Mobile phase A: water with 0.01% acetic acid; mobile phase B: isopropanol:acetonitrile (1:1, vol/vol). Sample tray temperature: 4°C; injection volume: 2 μL. An Orbitrap Exploris 120 mass spectrometer controlled by Xcalibur software (version 4.4, Thermo) was used for MS1 and MS2 data acquisition. Parameters: sheath gas flow rate: 50 Arb; aux gas flow rate: 15 Arb; capillary temperature: 320°C; full MS resolution: 60,000; MS/MS resolution: 15,000; collision energy: stepped NCE 20/30/40; spray voltage: 3.8 kV (positive) or −3.4 kV (negative).

Raw data were converted to mzXML format using ProteoWizard (43). Peak alignment, retention time correction, and peak area extraction were performed using XCMS (44). Metabolite identification was performed using a local in-house database and public databases (Human Metabolome Database [HMDB] [http://www.hmdb.ca], METLIN [http://metlin.scripps.edu], MassBank [http://www.massbank.jp/], and mzCloud [https://www.mzcloud.org]). Metabolites were identified by matching retention time, molecular mass (mass error <10 ppm), MS/MS spectra, and collision energy. All identifications were manually verified. The identification confidence level was ≥ level 2. Quality control (QC) was assessed using QC samples comprising pooled equal-proportion aliquots of all experimental samples. Total ion chromatograms (TICs) of QC samples were overlapped for comparison, and Pearson correlation analysis was performed (correlation coefficient > 0.9 indicates good reproducibility). PCA analysis was applied to all samples and QC samples; smaller variation among QC samples indicates higher method stability and data quality.

After total sum normalization, multivariate analysis was performed using the R package ropls. Differentially expressed metabolites were selected based on the VIP values from the first two components of the OPLS-DA model and univariate *P*-values (45, 46). Criteria: VIP ≥ 1 and *P*-value < 0.05. Pearson correlation analysis was used to assess the relationship between variables.

### Enrichment analysis and data visualization

Differential expression analyses were performed for mRNA, proteins, and metabolites. GO and KEGG enrichment analyses for transcriptomic and proteomic data were conducted using GOSeq (v1.26.0) (47) and KOBAS (v3.03) (48), respectively. GSEA v3.0 (49, 50) was used to analyze carbohydrate metabolism-related pathways. KEGG pathway enrichment for differential metabolites was performed using hypergeometric testing. Nine-quadrant plots, GSEA plots, and KEGG enrichment visualizations were generated using the ggplot2 (v3.5.1) (51) R package.

### Fermentation assays

To evaluate the fermentation capabilities of the bacterial strains WT, Δ*mnmE*, and CΔ*mnmE*, we utilized the VITEK 2 Gram-Positive Identification card (VITEK 2 GP Test Kit) (BioMérieux, France) following the manufacturer’s instructions and previously established protocols (52). The turbidity of the bacterial suspensions was standardized to a range of 4.5 to 5.5 on the McFarland scale to ensure consistency across samples. Fermentation of 20 different carbohydrates was assessed under the specific conditions outlined in the manual. Results were interpreted based on color changes, with red indicating a positive reaction and yellow indicating a negative reaction. To ensure the reliability of the findings, each test was performed in triplicate.

### Assay of intracellular reactive oxygen species

ROS levels were quantified using a Reactive Oxygen Species Assay Kit (Solarbio, China). The WT, Δ*mnmE*, and CΔ*mnmE* strains were cultured to mid-exponential growth phase, harvested and washed three times with 1 mL PBS buffer. The cells were resuspended in 20 mM MnCl_2_ solution and incubated at 37°C for 1 h, then washed three times with PBS. Subsequently, the pellets were resuspended in 10 μmol/L H_2_DCFDA solution provided by the kit and incubated at 37°C in the dark for 30 min. After washing, the cells were resuspended in 1 mL PBS. Subsequently, 100 μL aliquots were transferred to a sterile 96-well plate. Fluorescence intensity (FI) was measured using a microplate reader (Envision, PerkinElmer, USA) with excitation and emission wavelengths set at 488 nm and 525 nm, respectively. Each sample was analyzed in at least three biological replicates to ensure data reliability.

### Assay of intracellular metal content

The WT, Δ*mnmE*, and CΔ*mnmE* strains were grown in THB medium to an OD_600_ of 0.6, after which MnCl₂ was added to the cultures to induce metal stress. Cultures were further incubated for 30 min, followed by harvesting of bacterial cells via centrifugation at 8,000 × *g* for 10 min at 4°C. Harvested cells were washed three times with PBS supplemented with 0.25 mM EDTA to chelate extracellular metal ions, and subsequently washed three additional times with PBS alone to remove residual EDTA. Cells were dried at 110°C to a constant weight, then weighed. Dried cell pellets were digested in 66% (vol/vol) nitric acid at 95°C for 6 h. Digested samples were diluted to a final nitric acid concentration of 2% (vol/vol) using deionized water. Intracellular metal concentrations were analyzed by inductively coupled plasma mass spectrometry (ICP-MS). Metal content was quantified as micrograms of metal per gram of dry cell weight (μg/mg dry weight). Three independent biological replicates were performed for each strain and treatment condition.

### RT-qPCR analysis

WT and Δ*mnmE* were grown to the exponential phase (6 h) in THB medium at 37°C in an incubator with 5% CO_2_, and the bacterial cells were harvested and washed three times with PBS. Subsequently, total RNA was extracted using the E.Z.N.A. Bacterial RNA Kit (Omega, USA) and quantified by measuring the absorbance ratio at 260 nm/280 nm. Following the manufacturer’s instructions, the total RNA was reverse-transcribed into cDNA using the PrimeScript FAST RT reagent Kit with gDNA Eraser (Takara, Japan). RT-qPCR analysis was performed on a QuantStudio 5 Real-Time PCR System (Applied Biosystems, USA) with TB Green Premix Ex Taq II (Tli RNaseH Plus) (Takara, Japan), the primer sequences are listed in Table S6. Thermal cycling followed a standard two-step PCR amplification protocol (40 cycles: 95°C for 30 s, 95°C for 5 s, and 60°C for 30 s). The relative FC in mRNA expression was calculated using the 2^−ΔΔCt^ method, with *GAPDH* as the reference gene.

### Analysis of tRNAs and their corresponding anticodons

Genomic data of tRNAs for three model organisms (*Escherichia coli* CFT073 [GenBank: AE014075.1], *Bacillus subtilis* DSM 10 [GenBank: CP120681.2], and *Saccharomyces cerevisiae* S288C [GenBank: GCA_000146045.2]) and *Streptococcus pneumoniae* NCTC7465 (GenBank: LN831051.1) and *S. suis* 05ZYH33 (GenBank: CP000407) were retrieved from the National Center for Biotechnology Information (NCBI) database. Analysis of tRNAs responsible for translating specific codons was conducted using annotation features provided by the tRNAscan-SE website (https://lowelab.ucsc.edu/tRNAscan-SE/index.html). The gene-specific codon usage (GSCU) patterns for the two-family codon (NNA, NNG) and codon enrichment analysis were calculated, as previously described (30).

### Computational analysis of gene-specific codon usage patterns

The GSCU in the genome sequence of *S. suis* 05ZYH33 (GenBank: CP000407) was analyzed as previously described (30). Briefly, the codon frequency for each gene was compared against a background distribution. This distribution was simulated by randomly sampling a gene (with replacement) 1,000 times from all coding sequences (CDSs) in the genome to represent the random codon usage across the genome. For a target gene, its observed frequency of a specific codon (e.g., GAA) was compared against the distribution of frequencies from these 1,000 random samplings. The *P*-value was defined as the proportion of random samplings that yielded a frequency greater than or equal to the observed frequency. Subsequently, the *q*-value was obtained by false discovery rate (FDR) correction of all gene *P*-values using the *q*-value package in R. In this study, we used a threshold of *q*-value < 0.05 to define genes significantly enriched for specific U_34_codons (GAA/GAG, CAA/CAG). The value <0.05 to define genes significantly enriched foset of specific codons of interest (e.g., all GAA and CAA codons) in a gene, divided by the total number of all codons in that gene. The thresholds for defining enrichment (GAA/CAA > 0.0741, GAG/CAG > 0.0393) were the sum of its frequency based on the genome-wide distribution.

### Statistical analysis

Statistical analyses were performed using GraphPad Prism 8 software (GraphPad Software, CA, USA). All tests were repeated at least three times. *P* < 0.05 was considered statistically significant. All values are expressed as standard errors of the mean.

## Data Availability

The raw analysis data of transcriptomic have been deposited in Array-ExpressArrayExpress (EMBL-EBI; https://www.ebi.ac.uk/arrayexpress/) and are accessible through accession number E-MTAB-15431. The raw proteomic data have been deposited in the iProX (https://www.iprox.cn//page/project.html?id=IPX0016571000) repository under accession number PXD076839.

## SUPPLEMENTAL MATERIAL

Supplemental Figures spectrum.01201-26-s0001.pdf Fig. S1 to S6.

Table S1 spectrum.01201-26-s0002.xlsx Sequences of primers used in this study.

Table S2 spectrum.01201-26-s0003.xlsx Transcriptional responses in *S. suis* strains 05ZYH33 and Δ*mnmE*.

Table S3 spectrum.01201-26-s0004.xlsx Proteomic responses in *S. suis* strains 05ZYH33 and Δ*mnmE*.

Table S4 spectrum.01201-26-s0005.xlsx Metabolic responses in *S. suis* strains 05ZYH33 and Δ*mnmE*.

Table S5 spectrum.01201-26-s0006.xlsx Differentially expressed virulence factors.

Table S6 spectrum.01201-26-s0007.xlsx Primers for RT-qPCR analysis.

ASM does not own the copyrights to Supplemental Material that may be linked to, or accessed through, an article. The authors have granted ASM a non-exclusive, world-wide license to publish the Supplemental Material files. Please contact the corresponding author directly for reuse.
