# Supplementary material for: Multi-omics analysis identifies a role for Streptococcus suis MnmE in growth regulation and environmental adaptation
Source: Microbiol Spectr. 2026 May 22;14(7):e01201-26. doi: 10.1128/spectrum.01201-26 (PMC13340040; doi:10.1128/spectrum.01201-26)
Supplement: Supplemental Figures — Fig. S1 to S6. [file spectrum.01201-26-s0001.pdf]

Supplemental Figures

A

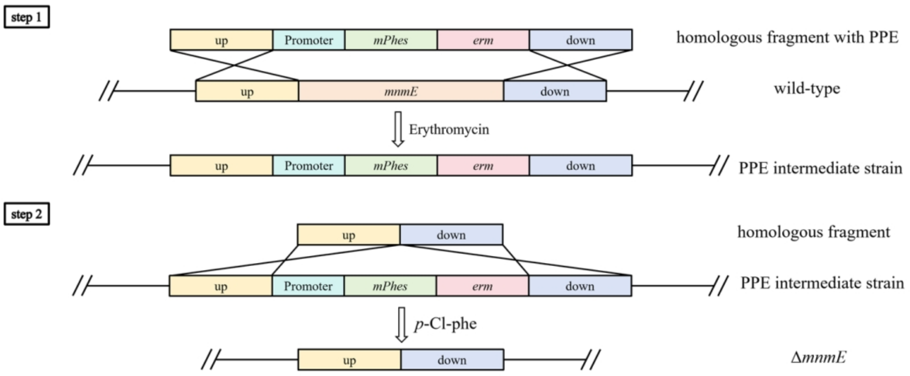

B

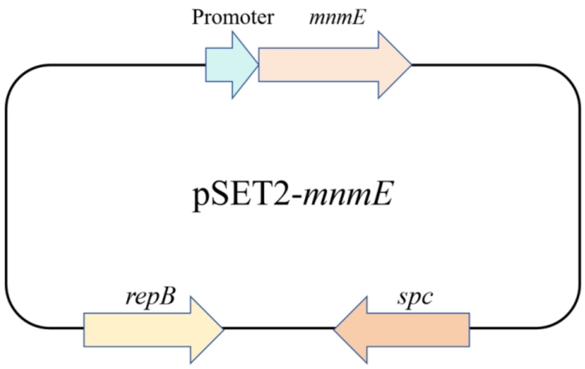

Fig. S1. Diagram illustrating the *mnmE* gene knockout and plasmid pSET2-*mnmE*. **(A)** Diagram for the two-step markerless gene-deletion strategy in this study, using *erm* (encoding an erythromycin-resistance) as a positive-selection marker, and *mPheS* (a conditionally lethal mutant allele of *pheS*, which encodes the α-subunit of phenylalanyl-tRNA synthetase (PheS), as a counter selectable marker (CSM). PPE = Promoter + *mPheS* + *erm*, *p*-Cl-phe = 4-Chloro-DL-phenylalanine. **(B)** Diagram of the plasmid pSET2-*mnmE* used for constructing the complemented strain. repB = replication protein B, *spc* = Spectinomycin resistance.

Fig. S2

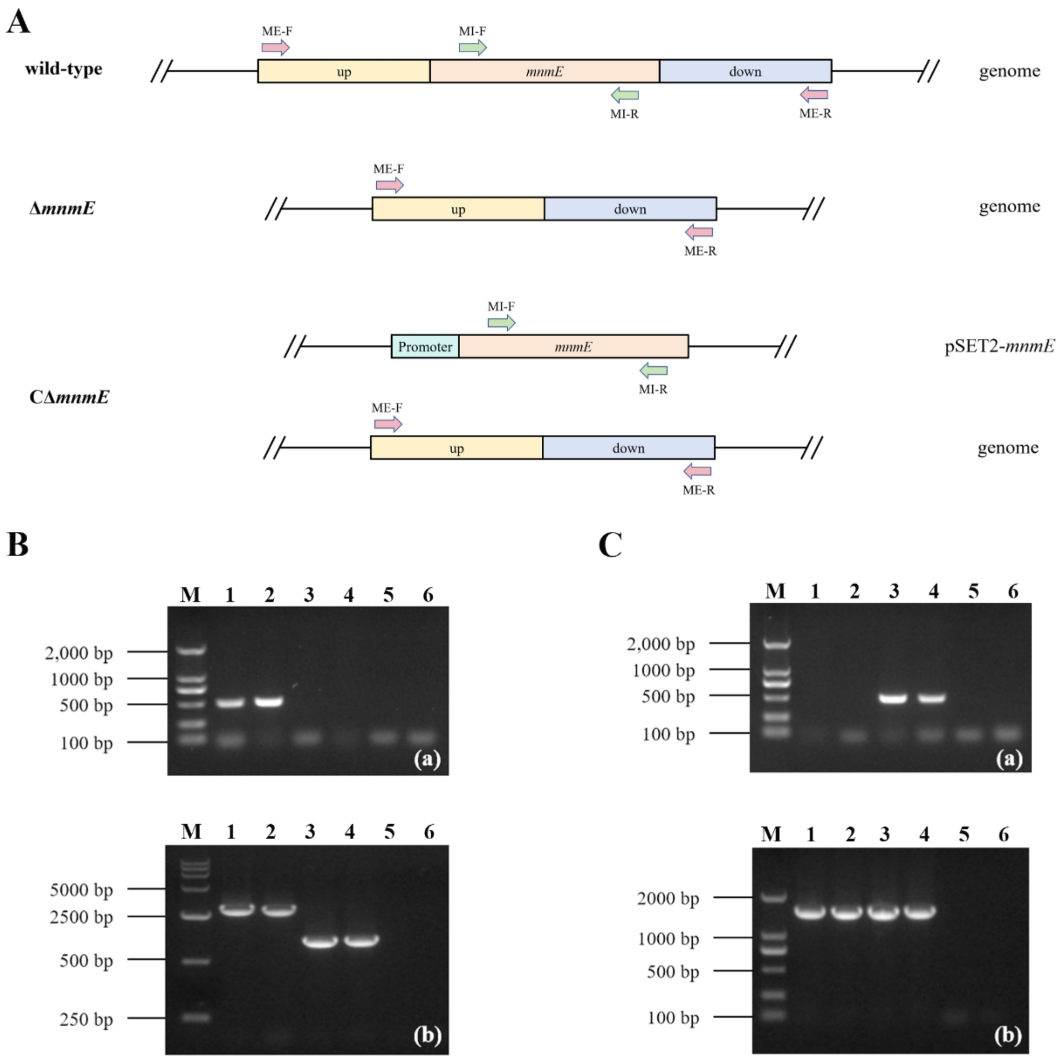
A
ME-F
MI-F
wild-type
up
mnmE
down
genome
MI-R
ME-R
ΔmnmE
ME-F
up
down
genome
ME-R
MI-F
Promoter
mnmE
pSET2-mnmE
MI-R
CΔmnmE
ME-F
up
down
genome
ME-R
B
M 1 2 3 4 5 6
2,000 bp
1000 bp
500 bp
100 bp
(a)
M 1 2 3 4 5 6
5000 bp
2500 bp
500 bp
250 bp
(b)
C
M 1 2 3 4 5 6
2,000 bp
1000 bp
500 bp
100 bp
(a)
M 1 2 3 4 5 6
2000 bp
1000 bp
500 bp
100 bp
(b)

Fig. S2. Verification of the *mnmE* gene deletion (Δ*mnmE*) and complementation (CΔ*mnmE*) strains.

(A) Diagram illustrating the identification of primer binding sites. Amplification of an internal fragment of the *mnmE* gene using primers MI-F/MI-R. Amplification of the *mnmE* gene flanking regions (including upstream and downstream sequences) using primers ME-F/ME-R. Using primers MI-F/MI-R, the WT and CΔ*mnmE* should yield a 500 bp amplicon, whereas Δ*mnmE* produces no band. Using ME-F/ME-R, the WT generates a 2,914 bp product, while Δ*mnmE* and CΔ*mnmE* yield a 1,540 bp band. **(B)** PCR verification of the Δ*mnmE* strain. **(a)** Using primers MI-F/MI-R. **(b)** Using primers ME-F/ME-R. *Lanes 1 and 2:* Genomic DNA templates from strains 05ZYH33 (wild-type); *Lanes 3 and 4*: Genomic DNA templates from Δ*mnmE* mutant; *Lanes 5 and 6*: Negative control (no template DNA). **(C)** PCR verification of the CΔ*mnmE* complementation strain. **(a)** Using primers MI-F/MI-R. **(b)** Using primers ME-F/ME-R. *Lanes 1 and 2:* Genomic DNA templates from strains Δ*mnmE*; *Lanes 3 and 4*: Genomic DNA templates from CΔ*mnmE*; *Lanes 5 and 6*: Negative control (no template DNA).

Fig. S3

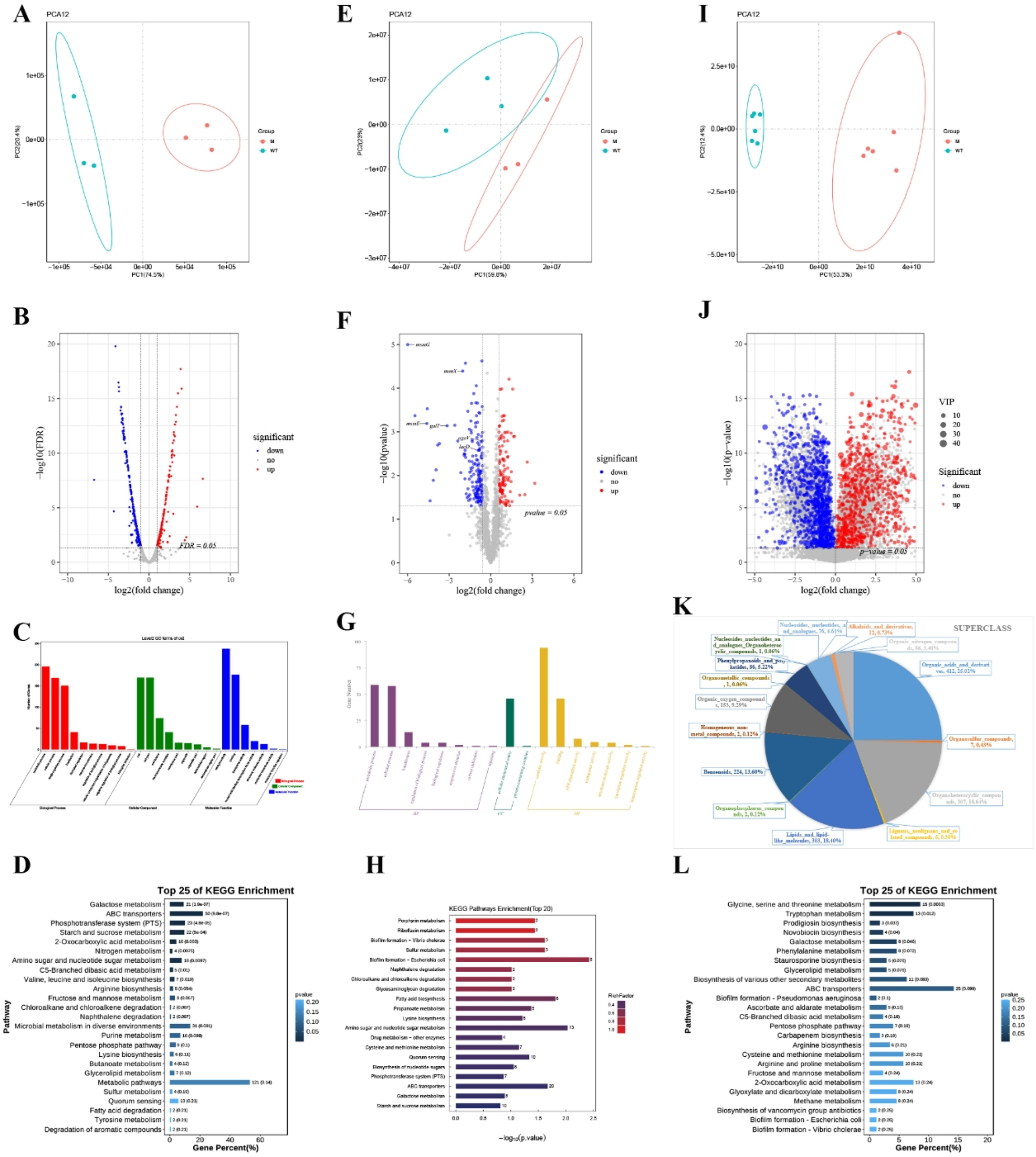
A
B
C
D
E
F
G
H
I
J
K
L
PCA12
significant
SUPERCLASS
Top 25 of KEGG Enrichment
KEGG Pathways Enrichment(Top 20)
Gene Percent(%)
Pathway
log2(fold change)
−log10(FDR)
−log10(pvalue)
−log10(p−value)
FDR = 0.05
pvalue = 0.05
p−value = 0.05
VIP
Significant

Fig. S3. Integrated multi-omics analysis of *S. suis* strains 05ZYH33 and Δ*mnmE*.

(A) Principal component analysis (PCA) of global transcriptome profiles. **(B)** Volcano plot of differentially expressed genes (DEGs) [adjusted $p < 0.05$, $|\log_2 FC| > 1$] between WT and Δ*mnmE*. **(C)** Gene Ontology (GO) functional classification of DEGs. **(D)** KEGG pathway enrichment analysis of DEGs. **(E)** PCA of global proteome profiles. **(F)** Volcano plot of differentially expressed proteins (DEPs) [adjusted $p < 0.05$, $|FC| > 1.5$] between WT and Δ*mnmE*. (Up-regulated: FC > 1.5; Down-regulated: FC < 0.67). **(G)** GO functional classification of DEPs. **(H)** KEGG pathway enrichment analysis of up-regulated DEPs. **(I)** PCA of global metabolome profiles. **(J)** Volcano plot of differential metabolites (DMs) [VIP ≥ 1, $p < 0.05$] between WT and Δ*mnmE*. **(K)** Functional classification of DMs (donut chart). **(L)** KEGG pathway enrichment analysis of DMs.

Fig. S4

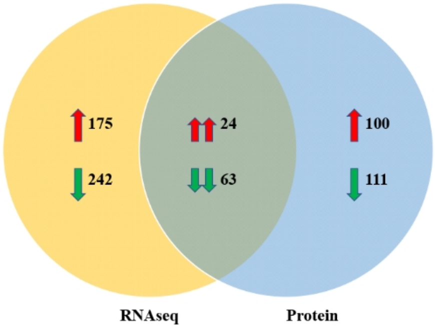

Fig. S4 Venn diagram of DEGs and DEPs. 17.3 % (87/504) of the DEGs showed concordant regulation at the protein level, with 24 co-upregulated and 63 co-downregulated.

Fig. S5

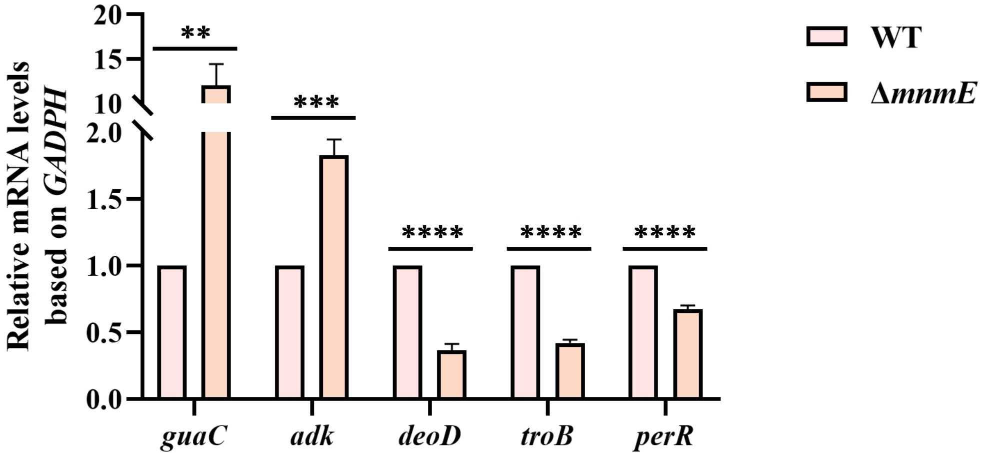

Relative mRNA levels based on GADPH
20
15
10
2.0
1.5
1.0
0.5
0.0
**
***
****
****
****
guaC
adk
deoD
troB
perR
WT
ΔmnmE

Fig. S5. RT-qPCR analysis for DEGs. The reliability of the transcriptomic data was verified by qRT-PCR. Several genes identified as significantly up- or down-regulated were randomly selected, and their expression was quantified with *GAPDH* serving as the internal reference gene.

Fig. S6

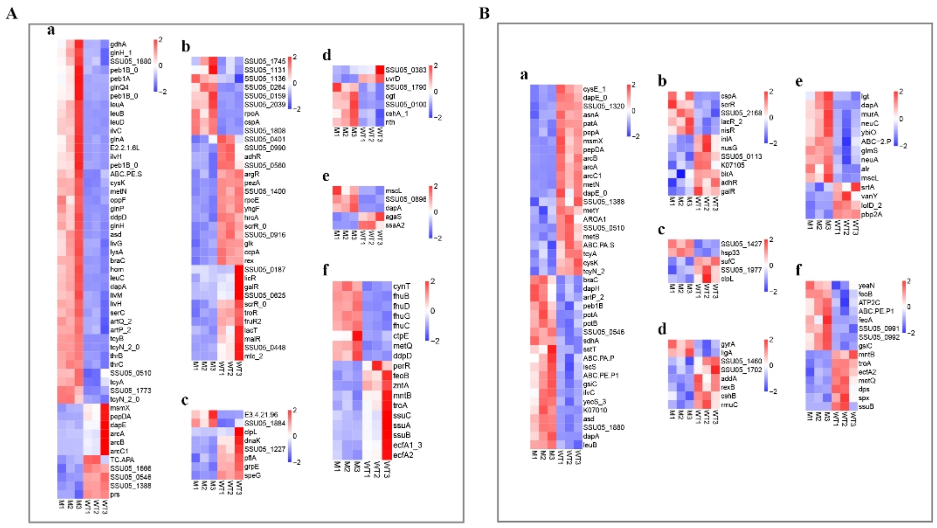
A
a
gdhA
glnH_1
SSU05_1880
peb1B_0
peb1A
glnQ4
peb1B_0
leuA
leuB
leuD
ilvC
glnA
E2.2.1.6L
ilvH
peb1B_0
ABC.PE.S
cysK
metN
oppF
glnP
ddpD
glnH
asd
livG
lysA
braC
hom
leuC
dapA
livM
livH
serC
artQ_2
artP_2
tcyB
tcyN_2_0
thrB
thrC
SSU05_0510
tcyA
SSU05_1773
tcyN_2_0
msmX
pepDA
dapE
arcA
arcB
arcC1
TC.APA
SSU05_1666
SSU05_0546
SSU05_1388
prs
b
SSU05_1745
SSU05_1131
SSU05_1136
SSU05_0264
SSU05_0159
SSU05_2039
rpoA
cspA
SSU05_1808
SSU05_0401
SSU05_0990
adhR
SSU05_0580
argR
pezA
SSU05_1400
rpoE
yhgF
hrcA
scrR_0
SSU05_0916
glk
ccpA
rex
SSU05_0187
licR
galR
SSU05_0625
scrR_0
troR
fruR2
lacT
malR
SSU05_0448
mlc_2
c
E3.4.21.96
SSU05_1884
clpL
dnaK
SSU05_1227
pflA
grpE
speG
d
SSU05_0383
uvrD
SSU05_1790
ogt
SSU05_0100
cshA_1
nth
e
mscL
SSU05_0898
dapA
agaS
ssaA2
f
cynT
fhuB
fhuD
fhuG
fhuC
ctpE
metQ
ddpD
perR
feoB
zntA
mntB
troA
ssuC
ssuA
ssuB
ecfA1_3
ecfA2
M1 M2 M3 WT1 WT2 WT3
2 0 −2
B
a
cysE_1
dapE_0
SSU05_1320
asnA
patA
pepA
msmX
pepDA
arcB
arcA
arcC1
metN
dapE_0
SSU05_1388
metY
AROA1
SSU05_0510
metB
ABC.PA.S
tcyA
cysK
tcyN_2
braC
dapH
artP_2
peb1B
potA
potB
SSU05_0546
sdhA
sstT
ABC.PA.P
iscS
ABC.PE.P1
gsiC
ilvC
yecS_3
K07010
asd
SSU05_1880
dapA
leuB
b
cspA
scrR
SSU05_2168
lacR_2
nisR
inlA
nusG
SSU05_0113
K07105
birA
adhR
galR
c
SSU05_1427
hsp33
sufC
SSU05_1977
clpL
d
gyrA
ligA
SSU05_1460
SSU05_1702
addA
rexB
cshB
rmuC
e
lgt
dapA
murA
neuC
ybiO
ABC−2.P
glmS
neuA
alr
mscL
srtA
vanY
lolD_2
pbp2A
f
yeaN
feoB
ATP2C
ABC.PE.P1
feoA
SSU05_0991
SSU05_0992
gsiC
mntB
troA
ecfA2
metQ
dps
spx
ssuB

Fig. S6. Heatmaps of selected COG functional classifications for DEGs) and DEPs.

(A) DEGs. (B) DEPs. (a) Amino acid transport and metabolism. **(b)** Transcription. **(c)** Post-translational modification, protein turnover, chaperones. **(d)** Replication, recombination and repair **(e)** Cell wall/membrane/envelope biogenesis. **(f)** Inorganic ion transport and metabolism
